# Arbuscular Mycorrhizal Fungi Restored the Saline–Alkali Soil and Promoted the Growth of Peanut Roots

**DOI:** 10.3390/plants12193426

**Published:** 2023-09-28

**Authors:** Dunwei Ci, Feifei Qin, Zhaohui Tang, Guanchu Zhang, Jialei Zhang, Tong Si, Jishun Yang, Yang Xu, Tianyi Yu, Manlin Xu, Kang He

**Affiliations:** 1 Shandong Peanut Research Institute, Qingdao 266100, China; cdw_2007@126.com (D.C.); jialing_300@163.com (F.Q.); guanchuzhang@126.com (G.Z.); xy52120092661@163.com (Y.X.); xumanlin@126.com (M.X.); 2 Shandong Academy of Agricultural Sciences, Jinan 250100, China; tangzhaohui1116@163.com (Z.T.); zhangjialei19@163.com (J.Z.); 3 College of Agronomy, Qingdao Agricultural University, Qingdao 266109, China; nmst12@163.com

**Keywords:** arbuscular mycorrhizal fungi, peanuts, saline–alkali soil, soil enzyme activity, soil nutrient

## Abstract

Peanut (*Arachis hypogaea* L.) is an important oil and cash crop. An efficient utilization of saline–alkali soil resources, the development of peanut planting in saline–alkali soil, and obtaining high and stable yield have become urgent needs to ensure peanut production. Arbuscular mycorrhizal fungi (AMF) have been reported to develop the potential productivity of host plants and improve their salt resistance and tolerance. However, there is still limited research on promoting the growth and morphology of peanut roots. Therefore, in this study, seeds of salt-tolerant peanut variety “HY 25” were coated with commercial AMF inoculant before being planted in saline–alkali and normal soils to investigate the effects of AMF on peanut root growth and rhizosphere soil. The results showed that root morphological characteristics were significantly increased by the use of AMF at the podding stage in saline–alkali soil and from the flowering and pegging stage to the maturity stage in normal soil. Of note, the total root volume of peanut inoculated with AMF significantly increased by 31.57% during the podding stage in saline–alkali soil. Meanwhile, AMF significantly increased the phosphatase and invertase activities in the peanut rhizosphere of saline–alkali soil from the flowering stage to maturity stage and soil CAT activity at the maturity stage (41.16~48.82%). In normal soil, soil phosphatase and urease activities were enhanced by AMF at the flowering stage and the podding stage, respectively. AMF also increased the contents of soil organic matter, available phosphorus, and hydrolysable nitrogen, but decreased soil EC in saline–alkali soil. In addition to the significant increase in soil available phosphorus content, AMF had no significant effect on the physical and chemical properties of the soil and other soil nutrients in normal soil. AMF significantly increased pod biomass and yield in saline–alkali soil and normal soil, and improved their agronomic characteristics. In conclusion, peanut seeds coated with AMF improved the root morphological characteristics of peanuts and improved the physical and chemical properties in peanut rhizosphere, especially in saline–alkali soil. The process of rhizosphere soil nutrient transformation was also enhanced. Finally, AMF improved plant agronomic traits to increase the pod yield (16.5~21.9%). This study provides the theoretical basis and technical support for the application of AMF in peanut production in saline–alkali soil.

## 1. Introduction

Peanut is an important oil and economic crop [1]. Unreasonable agricultural irrigation and population growth have exacerbated the contradiction between agricultural production and ecological health. Due to limited arable land resources and the need for regional agricultural development, peanut cultivation has begun in saline–alkali areas, such as coastal areas. Peanut is a moderately salt-tolerant crop with characteristics including drought resistance and barren tolerance [2]. However, soil salinization seriously affects peanut emergence, plant growth, and production efficiency [3]. Therefore, accelerating the utilization and improvement of saline–alkali land to develop peanut production in saline–alkali soil can improve the agricultural planting structure of saline–alkali soil and ensure the safe supply of grain and oil.

Arbuscular mycorrhizal fungi (AMF) is a type of fungi commonly found in soil, capable of forming reciprocal symbiosis with 90% of plant roots [4,5]. Utilizing the reciprocal symbiotic relationship between plants and AMF to improve crop productivity in saline soil is one of the new technologies used for improving saline–alkali soil [6]. Previous studies have shown that arbuscular mycorrhizal fungi can enhance plant growth in saline–alkali soils and restore vegetation [7,8]. When the host plant is subjected to salt stress, AMF can alleviate the damage of salt stress by strengthening the physiological response of the host plant to salt, such as by increasing the absorption of nitrogen, phosphorus, and potassium, reducing the content of Na^+^ and Cl^−^ in the host plant, and finally increasing the biomass of the host plant under salt stress [9,10,11,12]. AMF can also promote plant salt resistance by increasing the host plant root system’s activity, including expanding the root absorption area and changing root morphology, coordinating root hormone levels, which is beneficial for promoting root growth [13,14]. In addition, AMF can activate soil nutrients and alleviate the damage caused to plants by improving the soil’s microenvironment by, for example, improving the structure and richness of soil microbial communities, etc. [8,15].

Previous studies have found that single-applied AMF or its combined application with Ca can improve peanut biomass, yield, and peanut kernel quality in saline–alkali and normal soils [15]. The analysis found that applied AMF and Ca improved the diversity of the microbial community’s structure and enzyme activity in peanut rhizosphere soil in saline–alkali soil, especially increasing the abundance of Proteobacteria and Firmicutes. At the same time, it was found that *Sphingomonas* was the dominant genus, which improved peanut salt tolerance and promoted peanut growth and development [16]. Although there have been certain reports proving the effect of AMF in improving soil, promoting crop growth, and enhancing resistance [12], the impact of AMF on root development and the rhizosphere soil environment of peanuts in saline–alkali soil still needs further exploration.

Therefore, this experiment used peanut AMF seed coating technology to plant peanuts in saline–alkali soil and normal soil. By measuring the root system indicators and yield-related indicators of peanuts at different growth stages, as well as the physicochemical properties of rhizosphere soil, the ultimate goal was to evaluate the promoting effect of AMF on peanut yield.

## 2. Results

### 2.1. Effect of AMF on the Morphological Characteristics of Peanut Root System

**Figure 1:**
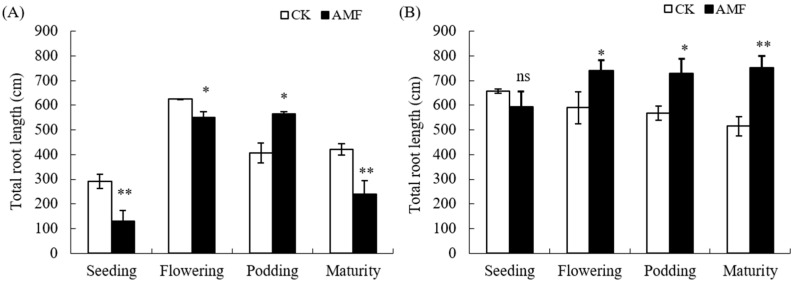
Influence of AMF on total root length of peanut in saline–alkali and normal soils. (**A**): GR saline–alkali soil, (**B**): LX normal soil. ns indicates non-significance; * indicates significant differences at *p* ≤ 0.05; ** indicates significant differences at *p* ≤ 0.01; data were expressed as mean ± SD (*n* = 3).

AMF significantly affected the total root length of peanuts under two soil types (Figure 1). On saline–alkali soil, AMF only significantly increased the total root length of the peanut during the podding stage (an increase of 27.82%) but significantly reduced the total root length of peanut during the seedling stage, flowering stage, and maturity stage. On normal soil, AMF significantly increased the total root length from flowering needle to maturity, with increases of 20.20%, 21.92%, and 31.24%, respectively. However, AMF had no significant effect on the total root length of peanut seedlings.

**Figure 2:**
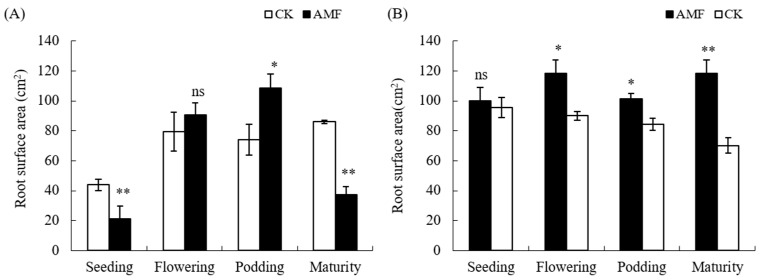
Influence of AMF on root surface area of peanut in saline–alkali and normal soils. (**A**): GR saline–alkali soil, (**B**): LX normal soil. ns indicates non-significance; ns indicates non-significance; * indicates significant differences at *p ≤* 0.05; ** indicates significant differences at *p ≤* 0.01; data were expressed as mean ± SD (*n* = 3).

The trend of AMF on the total surface area of peanut roots is consistent with the total root length (Figure 2), where AMF significantly increased the total surface area of peanut roots during the podding stage of saline–alkali soil, while the total surface area of peanut roots during the seedling and maturing stages showed opposite trends. There was no significant difference in the total surface area of root during the flowering period. On normal soil, the total root surface area of peanuts treated with AMF from the flowering stage to the maturity stage increased by 23.86%, 16.87%, and 40.86% compared to the treatment without AMF, respectively. The differences between the treatments were significant, but the differences in total root surface area during the seedling stage were not significant.

**Figure 3:**
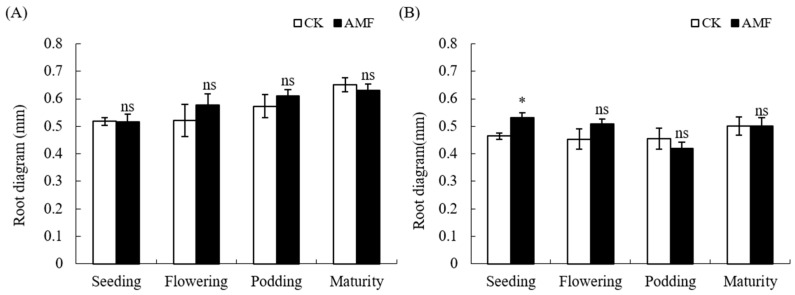
Influence of AMF on root diagram of peanut in saline–alkali and normal soils. (**A**): GR saline–alkali soil, (**B**): LX normal soil. ns indicates non-significance; * indicates significant differences at *p ≤* 0.05; data were expressed as mean ± SD (*n* = 3).

Interestingly, AMF only significantly increased the root diameter of peanuts at the seedlings stage in normal soil, but had no significant effect on root diameter from the flowering stage to maturity stage. Likewise, the effect of AMF on the root diameter of peanuts grown in saline–alkali soil during the entire growth period was not significant (Figure 3).

**Figure 4:**
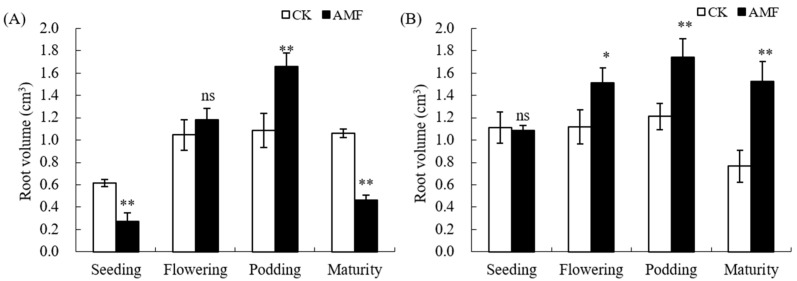
Influence of AMF on root volume of peanut in saline–alkali and normal soils. (**A**): GR saline–alkali soil, (**B**): LX normal soil. ns indicates non-significance; * indicates significant differences at *p ≤* 0.05; ** indicates significant differences at *p ≤* 0.01; data were expressed as mean ± SD (*n* = 3).

The impact trend of AMF on the total root volume of peanuts in saline–alkali soil and normal soils is similar to that of total root length (Figure 4). AMF significantly increased the total root volume of peanuts in normal soils during the flowering stage, podding stage, and maturity stage, with an increase of 25.94%, 30.30%, and 49.69% in the three stages, respectively. However, the total root volume of peanuts inoculated with AMF in saline- alkali soil only significantly increased during the podding stage (*p* ≤ 0.01), with an increase of 31.57%.

Based on the above analysis, the AMF coating of peanut seeds mainly promotes the root growth of peanut podding stage in saline–alkali soil, while it has a certain promoting effect on the root growth of peanuts from the flowering stage to the maturity stage in normal soil.

### 2.2. Effect of AMF on Enzyme Activity in Rhizosphere Soil

**Figure 5:**
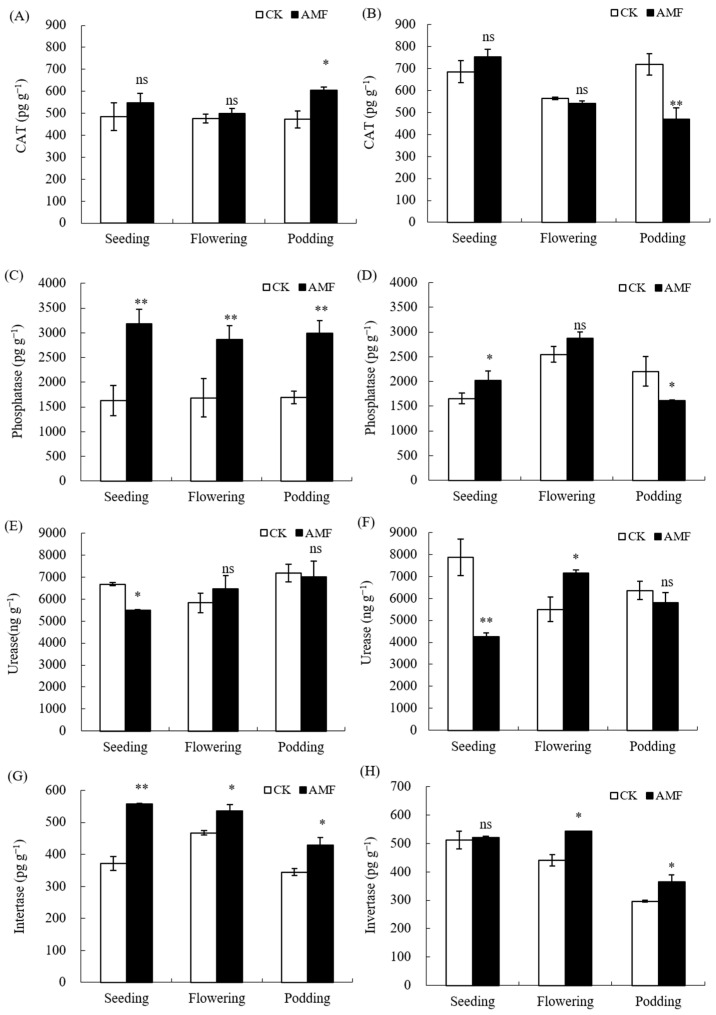
Influence of AMF on soil enzyme activities in peanut rhizosphere in saline–alkali and normal soils. (**A**,**C**,**E**,**G**): GR saline–alkali soil, (**B**,**D**,**F**,**H**): LX normal soil. ns indicates non-significance; * indicates significant differences at *p ≤* 0.05; ** indicates significant differences at *p ≤* 0.01; data were expressed as mean ± SD (*n* = 3).

The soil enzyme plays an important role in the soil’s nutrient metabolism and cycling. Therefore, this experiment analyzed the enzyme activities in the rhizosphere soil during the seeding stage and flowering stages, as well as the podding stages. AMF had no significant effect on catalase (CAT) activity in the rhizosphere soil during the seeding and flowering stages in saline–alkali and normal soils (Figure 5). AMF significantly increased CAT activity in the peanut rhizosphere soil during the podding stage in saline–alkali soils, On the contrary, AMF significantly decreased CAT activity in normal soil (Figure 5B). AMF significantly increased the phosphatase activity in the rhizosphere soil of peanut from seeding to podding stage in saline–alkali soil, with an increase of 48.82%, 41.16%, and 43.38%, respectively. However, on normal soil, AMF only significantly increased the phosphatase activity during the flowering stage, and significantly reduced the phosphatase activity during the mature stage. Meanwhile, AMF also significantly increased the urease activity of peanuts during the flowering stage in saline–alkali and normal soils but decreased the urease activity of peanuts during the podding stage. In addition, AMF significantly increased the soil invertase activity in the peanut rhizosphere during the seeding and podding stages of normal soil, with an increase of 18.83% and 18.75%, respectively. The effect of AMF on soil sucrase in saline–alkali soil is greater than that in normal soil, which was significantly increased by 33.35%, 12.70%, and 19.84% from the seeding stage to the podding stage.

### 2.3. Impact of AMF on Soil Chemical Properties

**Figure 6:**
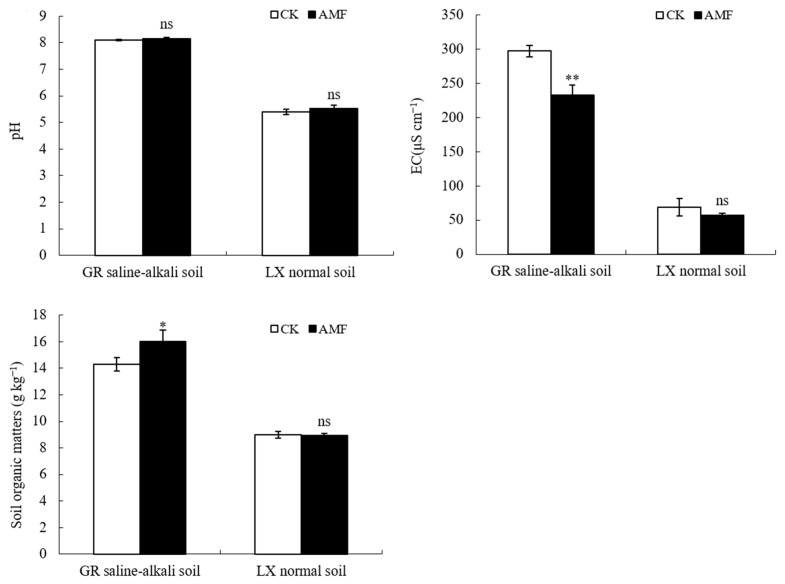
Influence of AMF on soil pH, EC and soil organic matters in peanut rhizosphere at mature stage in saline–alkali and normal soils. ns indicates non-significance; * indicates significant differences at *p ≤* 0.05; ** indicates significant differences at *p ≤* 0.01; data were expressed as mean ± SD (*n* = 3).

In saline–alkali soil, the electrical conductivity of the rhizosphere soil treated with AMF significantly decreased by 21.57% compared to untreated soil, and the soil organic matter content significantly increased by 10.66%. However, the applied AMF did not significantly affect the pH, electrical conductivity, and organic matter of the peanut rhizosphere soil in normal soil (Figure 6). 

**Figure 7:**
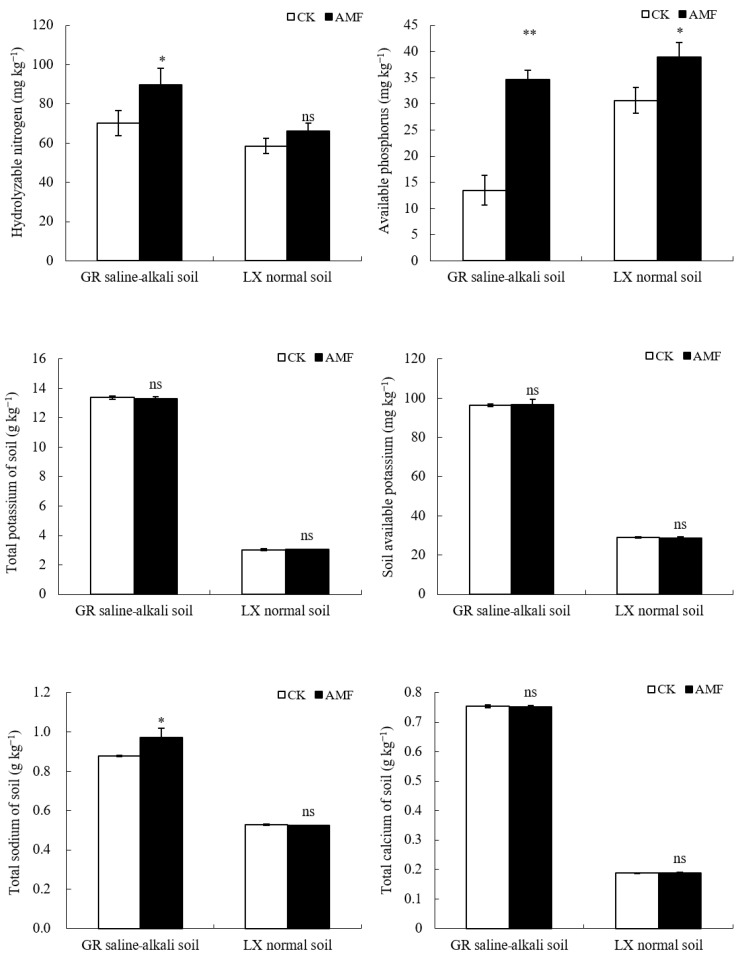
Influence of AMF on nutrient ions in peanut rhizosphere at mature stage in saline–alkali and normal soils. ns indicates non-significance; * indicates significant differences at *p ≤* 0.05; ** indicates significant differences at *p ≤* 0.01; data were expressed as mean ± SD (*n* = 3).

Similarly, AMF significantly increased the content of alkali hydrolyzed nitrogen and total sodium of the peanut rhizosphere in saline–alkali soil, with increases of 21.54% and 9.71%, respectively. It significantly increases the soil available phosphorus content by 60.98%. However, AMF had no significant effect on the content of available potassium, total potassium, and total calcium in saline–alkali soil. On normal soil, applied AMF only significantly increased the soil available phosphorus content (increase of 21.21%), and there was no significant difference in the soil’s available nitrogen, available potassium, total potassium, total sodium, and total calcium content (Figure 7).

### 2.4. Effect of AMF on Agronomic Characteristics and Yield of Peanuts

AMF treatment significantly increased the number of primary branches, secondary branches, and 10 cm nodes at the base of the first lateral branch of peanut in saline–alkali soil, and significantly increased the height, length, and number of leaves on the main stem (Table 1). 

However, in normal soil, AMF only significantly increased the number of secondary branches and the number of 10 cm nodes at the base of the first lateral branch, significantly reducing, in contrast, the height of the main stem and the length of the lateral branch, with no significant effect on the number of primary branches and the number of leaves on the main stem. AMF treatment significantly increased the peanut pod yield in saline–alkali and normal soils, with increases of 21.9% and 16.5%, respectively. In addition, AMF treatment significantly increased the kernel yield of peanuts in saline–alkali soil, but had no significant effect on the kernel yield of peanuts in normal soil (Table 2).

## 3. Discussion

### 3.1. Effect of AMF on the Morphological Characteristics of Peanut Root System

Arbuscular mycorrhizal fungi (AMF) can form mutualistic symbioses with 90% of plant roots, which usually have a positive effect on plant growth. Numerous studies have shown that inoculation with AMF can improve plant root growth and development [5,11,14,17,18]. The results of this experiment indicate that the AMF coating treatment of peanut seeds also has a significant effect on promoting the growth of peanut roots in normal soil (Figure 1 and Figure 2), but it shows an inhibitory effect on the growth of roots in the seedling and mature stages in saline–alkali soil. This indicates that compared to normal soil, saline–alkali soil not only affects the growth of AMF but also affects the establishment of arbuscular mycorrhizal symbiosis with peanut roots. Previous studies have shown that AMF can promote plant growth and restore vegetation on saline–alkali soils [6,7], but the soil salinity, in turn, affects the growth and development of arbuscular mycorrhizal fungi, further affecting their symbiosis relationship with plants. A study has found that under salt stress conditions, the infection rate of AMF in host plants is extremely low and there are only hyphae without arbuscular structure after being inoculated with AMF for 2.5 months [7]. Different strains of AMF have different tolerances to saline–alkali stress, and an increase in soil salt concentration usually affects AMF spore germination, shortens the bud tube elongation, inhibits mycelial branching, and thus reduces the chance of AMF initial infection [6,10,12]. In addition, the growth of roots and hyphae is inhibited by saline–alkali stress, which reduces the ability of extracellular hyphae to infect again [10]. Other studies have shown that on normal soil, 50-day-old maize inoculated with AMF inhibited root growth, including significant reductions in root length, surface area, volume, and tip number. However, as the plant grew, the effect is reversed [17]. Therefore, the inhibitory effect on the root of peanut in saline–alkali soil may be due to the inhibition of AMF growth and development under the saline–alkali soil environment. At the same time, when AMF initially established a symbiotic relationship with peanut, the growth of peanut was inhibited by saline–alkali stress and photosynthesis, and carbohydrates transferred to the roots were also reduced. AMF symbiotes produced a huge mycelial network, The growth and development of fungi leads to the absorption of a large amount of nutrients through seedling hosts, as fungi consume most of the photosynthetic products of seedlings, leading to carbon competition between AMF and peanut roots, thereby inhibiting the formation of mycorrhizal fungi and plant root growth [17,19]. AMF can promote plant growth and development under artificially controlled conditions, but its application in the field is affected by various environmental factors. For instance, with the invasion of indigenous mycorrhizal fungi, nutrient competition between mycorrhizal fungi intensifies, which may lead to the gradual disappearance of early advantages [20]. This study indicates that AMF can promote the growth and development of peanut roots during the podding stage, but has a significant inhibitory effect on the maturity stage (Figure 3 and Figure 4). The reason for this contradictory effect may be due to the competition between rhizobia infection and AMF, but this mechanism needs further research.

### 3.2. Effect of AMF on Enzyme Activity in Rhizosphere Soil

Arbuscular mycorrhizal fungi can enhance the saline–alkali tolerance of plants by establishing a symbiotic relationship so as to promote the growth of plants in saline–alkali soil. Previous studies have shown that the promotion of root morphology changes via the use of AMF is an important mechanism for improving plant salt tolerance [10,17]. Under natural saline soil and artificial salt stress, AMF increases maize root injury flow, enhances maize root vitality, and thus enhances maize salt tolerance [9]. Further research found that in the range of 0–2.0 g kg^−1^ salt concentration, AMF inoculation increased maize root activity, average root diameter, and total root volume; increased root length with diameters of 0.2–0.4 mm and 0.4–0.6 mm; prevented the transformation of root structure towards thicker roots; and thus alleviated the harm of salt stress on maize [13]. Under different salt concentrations, AMF inoculation significantly increased the total root length, surface area, volume, and lateral root quantity of Phoebe, promoting root growth [10,21]. The reason why AMF enhances plant salt tolerance may be due to the secretion of metabolic substances that stimulate plant root growth during the formation of mycorrhizal fungi. Similarly, the formation of mycorrhizal symbionts inhibits the activity of root meristem tissue, increases the number of adventitious and lateral roots, and enhances root vitality [11].

Soil enzymes are instrumental in the mineralization of soil organic matter and the material cycling of nutrients such as carbon, nitrogen, and phosphorus. Their activity provides an indication of the biochemical reactions taking place in the soil, which, in turn, affect the formation and accumulation of soil nutrients. Research has shown that inoculation with AMF enhances the activities of sucrase, catalase, and alkaline phosphatase in rhizosphere soil [5,22,23], and is also beneficial for the increase in alkaline phosphatase, urease, sucrase, cellulase, protease, and catalase activities in rhizosphere soil [10,11]. Similarly, previous studies have found that AMF enhances the activities of urease and sucrase in peanut rhizosphere soil, but has no significant effect on the activities of catalase and phosphatase [15]. The results of this experiment revealed that AMF significantly heightened the activities of phosphatase and sucrase in the rhizosphere soil of peanuts at various developmental stages, with a surge in catalase activity only during the maturity stage, while remaining neutral to urease activity. On normal soil, AMF augmented phosphatase activity during the flowering stage, urease activity during the podding stage, and sucrase activity during the podding and maturity stages (Figure 5). The disparate effects of AMF on enzyme activity in peanut rhizosphere soil between two experiments may be due to differences in the physicochemical properties of different saline–alkali soils.

The respiratory metabolism of soil organisms yields hydrogen peroxide, whose accumulation can have toxic effects on the organisms and soil. Catalase catalyzes the decomposition of hydrogen peroxide into water and oxygen, counteracting the toxic effect on soil. In this experiment, we observed that AMF promotes the activity of catalase in the rhizosphere soil of peanuts growing in saline–alkali soil during maturity stage (Figure 5A,B). This finding indicates that AMF can improve the detoxification of saline–alkali soil to some extent and alleviate the harm caused by saline–alkali stress to peanuts.

Soil phosphatase activity serves as an important indicator for evaluating the biological transformation of phosphorus in soil, which converts soil organic phosphorus into plant-available forms under the enzymatic action of phosphatase. Research has shown that the formation of AMF mycorrhizal fungi requires a certain amount of phosphorus. Under salt stress, the dependence of plants on AMF increases when the phosphorus demand of plants is not met [9], and root exudates of plants are enhanced, promoting the germination of AMF spores and the growth of mycelial, in addition to strengthening the symbiotic relationship between plants and AMF [6]. Our experimental results showed that AMF promotes an increase in available phosphorus content in the rhizosphere soil of peanuts in saline–alkali soil, and phosphatase activity in the rhizosphere increases through the flowering stage to the maturity stage of peanut growth and development, indicating that the symbiotic relationship between AMF and peanut is strengthened in saline–alkali soil (Figure 5C,D). AMF can cross the root surface area through hyphae and extend to the soil outside the root system to absorb phosphorus that cannot be absorbed by the plant root system, expanding the absorption range of available phosphorus, increasing the absorption of insoluble inorganic phosphorus, transporting and transferring available phosphorus from non-rhizosphere soil to the rhizosphere, and increasing the content of available phosphorus in the rhizosphere [9,10,15]. Additionally, AMF may stimulate phosphatase secretion in peanut rhizosphere soil, promote phosphatase activity, and more effectively promote the mineralization and bioavailability of organic phosphorus in peanut rhizosphere soil in saline–alkali soil, thereby promoting the effective absorption and utilization of phosphorus by peanut roots in saline–alkali soil. This may be an important factor for AMF to improve peanut phosphorus nutrition and enhance peanut salt tolerance [6,8]. In normal soil, in addition to the expanded absorption of available phosphorus by AMF hyphae, the increase in phosphatase activity (flowering stage) is also an important factor in promoting the content of available phosphorus in the rhizosphere.

Invertase is abundant in soil and plays a role in converting soil carbohydrates, as it catalyzes the hydrolysis of sucrose into glucose and fructose, effectively increasing the level of soluble nutrients in the soil. It is recognized as an important indicator of soil biological activity, fertility, and maturation [24]. Research has shown that invertase, being an enzyme for organic carbon decomposition, has activity that increases with microbial activity [24]. Microorganisms are able to accelerate the decomposition of soil organic carbon through the action of invertase, thereby providing nutrients for plants [6,25]. The results of this experiment demonstrated that AMF increased the activity of invertase in the rhizosphere of peanut from flowering to the maturity stage, both in saline–alkali and normal soils, indicating that AMF can enhance the soil biological activity and improve soil microbial status from peanuts’ flowering to the maturity stage. This occurs through the mechanism of increasing invertase activity, which catalyzes the mineralization rate of soil organic carbon, thereby providing a sufficient amount of carbon nutrients for peanuts (Figure 5G,H).

### 3.3. Impact of AMF on Soil Chemical Properties

In addition to phosphorus, AMF also has a significant impact on soil nitrogen content. Research has shown that AMF can increase the content of available nitrogen, ammonium nitrogen, nitrate nitrogen and other nutrients in the rhizosphere soil of *Paris polyphylla* seedlings, and can reduce the amount of available potassium [26]. AMF forms mycorrhizal symbiotes with host plants, which transform and absorb inorganic nitrogen, amino acids, and complex organic nitrogen through extracellular hyphae, and transport them to intracellular hyphae for further transformation into NH_4_^+^, which finally participates in plant nitrogen metabolism [24,27,28]. The results of this study found that AMF increased the alkaline nitrogen content of peanut rhizosphere in saline–alkali soil, but the key enzyme urease activity involved in soil nitrogen conversion was not affected by AMF, with no difference being observed in normal soil. This indicates that AMF may increase the rhizosphere’s nitrogen content by increasing peanut root activity and promoting the secretion of nitrogen-containing compounds into the soil by the roots [27], altering the nitrogen fixation ability of other soil microorganisms such as rhizobia [29], or, alternatively, promoting the mineralization of soil organic nitrogen and releasing a large amount of inorganic nitrogen through the secretion of its own extracellular hyphae [6,20].

Organic matter, as the main substrate and an important carrier of soil enzyme activity, has a significant positive correlation with its content and soil enzyme activity [20]. Research has shown that under soil salt stress, AMF can improve the salt tolerance of host plants by increasing the content of soil organic matter in the roots [25]. Within a certain range, the increase in soil organic matter content, in turn, has an important impact on AMF spore density and hyphal secretion [26,28]. The results of this study showed that AMF has a certain effect on increasing organic matter in peanut rhizosphere soil in saline–alkali soil, while the effect is not significant in normal soil (Figure 6). The relationship between various enzyme activities and organic matter content is consistent, indicating that the increase in organic matter in peanut rhizosphere may be one of the factors contributing to AMF’s improvement of peanut salt tolerance. Additionally, organic matter is the main source of soil organic carbon, and under the action of highly active soil enzymes, especially sucrase, it provides a more sufficient carbon source for the flowering, needle placement, and fruit development of peanuts in saline–alkali soil.

During the process of symbiosis establishment between AMF and plants, the roots breathe, secrete substances such as H^+^ and organic acids, and lower the soil’s pH [27]. In this experiment, AMF had no significant effect on the pH value of peanut rhizosphere soil in saline–alkali or normal soil. AMF reduced soil conductivity in saline–alkali soil, which is inconsistent with some research results reporting that inoculation with AMF significantly reduced the soil’s pH and increased its conductivity [26,30]. However, this reason may need verification in future experiments.

## 4. Materials and Methods

### 4.1. Test Materials

The experimental materials were selected from the salt-tolerant variety Huayu 25 (HY 25).

### 4.2. Overview of the Experimental Site and Basic Soil Characteristics

The experimental sites are located at Laixi Experimental Base (LX) of Shandong Peanut Research Institute and Guangrao Experimental Base (GR) of Shandong Academy of Agricultural Sciences. Laixi is located at longitude 120°12′–120°40′ E, latitude 36°34′–37°09′ N, and Guangrao is located at longitude 118°17′–118°57′ E and latitude 36°56′–37°21′ N. The soil type of the Laixi experimental base is tidal soil (normal soil), while the soil of the Guangrao experimental base is saline–alkali soil, falling into the category of moderate saline–alkali soil. The soil characteristics are shown in Table 3.

### 4.3. Test Methods

#### 4.3.1. Experimental Design

Peanuts are cultivated using ridge raising and film covering, with one row and two rows, with a ridge spacing of 85 cm, a row spacing of 30 cm, a hole spacing of 17.4 cm, and two seeds per hole. Before sowing, 975 kg · hm^−2^ of Hefei (N-P_2_O_5_-K_2_O, 15-15-15) and 600 kg · hm^−2^ of superphosphate (containing 18% P_2_O_5_ and 12% CaO) are applied as the base fertilizer. After soaking the seeds slightly, they are mixed and coated with arbuscular mycorrhizal fertilizer (AMF mycorrhizal fertilizer) at a ratio of 1 kg mycorrhizal fertilizer to 1 hm^2^ of the seed amount before sowing. This experiment used the treatment without AMF seed coating as the control. Other cultivation management practices shall follow the high-yield requirements of the field. Sowing was carried out on 15 May and harvesting on 15 September.

The tested AMF bacterial fertilizer is granular and provided by Norman Lear (Qingdao) Environmental Energy Technology Co., Ltd. (Qingdao, China) The AMF bacterial fertilizer used for inoculation is a 1:1 mixture of two bacterial strains, Funneliformis mosseae BEG95 and Rhizophagus irregularis SA. In our preliminary screening experiment, we found that this ratio is the best for peanut growth and development. The inoculum is made of sterile mixed clay, and 10 g of inoculum contains 500 spores [15].

#### 4.3.2. Sample Collection

The experiment was conducted on 42 days (late seedling stage), 57 days (flower needle stage), 84 days (pod stage), and 105 days (mature stage) after peanut sowing, respectively, to collect root samples. The method is as follows: Firstly, cut the aboveground parts and keep them fresh for future use. To avoid damage to the root system caused by excessive water pressure during direct flushing, place the divided soil layer on a specially designed steel sieve with a pore size of 1.00 mm. Carefully shake off the rhizosphere soil and pick out the root system inside the soil layer. Place it in an ice box, bring it back indoors, rinse it thoroughly, and then place it in a refrigerator for future use.

#### 4.3.3. Root System Determination

Use a scanner (model Epson7500, resolution 400 bpi) to scan the root system. When scanning, place the roots in a specially made transparent tray and add 3–5 mL of water to avoid an entanglement of the root branches. After scanning, analyze the saved image using the WinRhino Pro (5.0a) analysis program.

#### 4.3.4. Soil Sample Collection

On the 57th day (flower needle stage), 84th day (pod stage), and 105th day (mature stage) after peanut sowing, the S-type 5-point mixed sample method was used to collect mixed soil samples of 0–40 cm in the peanut root layer. After removing gravel, debris, and plant roots, the soil samples were divided into two parts. One part was placed in a sterile bag and stored at −20 °C for future use in soil enzyme activity. The other portion was air-dried, ground, and sieved to determine the soil’s physical and chemical indicators.

#### 4.3.5. Determination of Enzyme Activity in Rhizosphere Soil

The activities of soil catalase, phosphatase, urease, and sucrase were measured using the enzyme-linked immunosorbent double antibody sandwich method. The determination method and operating steps were carried out according to the soil catalase (CAT) ELISA detection kit, soil phosphatase ELISA detection kit, soil urease ELISA detection kit, and soil urease ELISA detection kit, respectively. The soil invertase ELISA detection kit (all kits provided by Qingdao Shenggong Biotechnology Co., Ltd, Qingdao, China) was operated according to the manufacturer’s instructions, and the OD value was measured using a TECAN enzyme labeling analyzer (Infinite F50, Männedorf, Switzerland) at a wavelength of 450 nm.

#### 4.3.6. Determination of Soil Physicochemical Properties

The soil pH value and conductivity were measured using a pH meter (5:1) and a conductivity meter, respectively. The soil was sieved through a 50 mesh sieve to determine soil organic matter, available phosphorus, and alkaline hydrolyzed nitrogen [31]. The soil organic matter was treated with a potassium dichromate external heating method. Alkaline hydrolyzed nitrogen was evaluated using the alkaline hydrolysis diffusion method [32]. The effective phosphorus was determined using a sodium bicarbonate extraction molybdenum antimony colorimetric method [33]. The soil was ground with a ball mill and sieved through a 100mesh sieve for the determination of total potassium, available potassium, total calcium, and total sodium in the soil. The determination of total potassium in soil follows the operating instructions of the soil total potassium content kit (provided by Qingdao Shenggong Biotechnology Co., Ltd, Qingdao, China), using the concentrated sulfuric acid perchloric acid high-temperature digestion flame photometer method for determination [32]. According to the operating instructions of the soil available potassium content kit (provided by Qingdao Shenggong Biotechnology Co., Ltd, Qingdao China), the ammonium acetate flame photometer method was used to measure soil available potassium. According to the instructions of the Soil Total Calcium Content Kit (provided by Qingdao Shenggong Biotechnology Co., Ltd, Qingdao China), the soil total calcium was measured using the concentrated sulfuric acid high-temperature digestion flame photometer method. The total sodium in soil was determined using the concentrated sulfuric acid high-temperature digestion flame photometer method according to the soil total sodium content test kit (provided by Qingdao Shenggong Biotechnology Co., Ltd, Qingdao China) [33].

#### 4.3.7. Determination of Plant Agronomic Traits and Pod Yield

During harvest, 10 representative plots were selected from each plot for continuous sampling, and variables including the main stem height, lateral branch length, number of branches, number of main stem leaves, number of 10 cm nodes at the base of the first pair of lateral branches, number of fruits per plant, number of full fruits, number of double kernels, weight of 100 fruits, weight of 100 kernels, and kernel yield were investigated. The method used is as follows: harvest two rows per plot of 2m for each treatment and weigh after natural air-drying and calculate the pod yield.

### 4.4. Data Statistics and Analysis

Microsoft Excel 2003 was used to organize and plot the data. All data in the graph were mean ± standard deviation (SE), and SPSS 16.0 was used for multivariate statistical analysis. One way ANOVA (Duncan test) was used for significance difference analysis.

## 5. Conclusions

This study utilized AMF coating on peanut seeds to promote root growth and development during the growth period of peanut in normal soil after flowering. By increasing the activities of phosphatase, urease, and sucrase in the rhizosphere soil of peanut at different development stages, the soil microenvironment was improved, thereby increasing pod yield. In saline–alkali soil, AMF coating primarily alters the root structure during the podding stage and increases the activities of catalase, phosphatase, and sucrase in the peanut rhizosphere. AMF coating also accumulates soil organic matter and nutrient ions such as carbon, nitrogen, and phosphorus; reduces electrical conductivity, thereby reducing the toxicity of saline–alkali soil, improving the soil microenvironment of saline–alkali soil; and increases the fertility of the rhizosphere soil, finally promoting the development of peanut growth and production.

## Figures and Tables

**Table 1:** Effects of arbuscular mycorrhizal fungi on agronomic characteristics of peanut in soil.

	Treatment	Stem Height (cm)	Lateral Branch Length (cm)	Primary Branches Number	Secondary Branches Number	Stem Leaf Number	Node Number of the 10 cm Base of Lateral Branches
GR Saline–alkali soil	CK	21.3 ± 2.7	24.3 ± 1.5	4.5 ± 0.3	3.5 ± 0.5	12.7 ± 1.3	5.7 ± 0.5
	AMF	32.9 ± 3.5 **	39.2 ± 2.5 **	5.2 ± 0.2 *	4.2 ± 0.3 *	14.8 ± 1.4 **	6.3 ± 0.4 *
LX Normal soil	CK	31.5 ± 4.2	32.8 ± 3.9	5.2 ± 0.6	3.7 ± 0.3	14.3 ± 0.3	4.7 ± 0.5
	AMF	27.7 ± 2.5 *	24.1 ± 2.9 **	5.2 ± 0.3 ^ns^	4.3 ± 0.4 *	14.2 ± 0.2 ^ns^	6.0 ± 0.8 *

^ns^ indicates non-significance; * indicates significant differences at *p ≤* 0.05; ** indicates significant differences at *p ≤* 0.01.

**Table 2:** Effects of arbuscular mycorrhizal fungi on yield and yield components of peanut in soils.

	Treatment	Yield (kg/hm^2^)	Total Pod Number	Full Pod Number	Number of Double Kernels	100-Pod Weight (g)	100-Kernel Weight (g)	Shelling Rate (%)
GR Saline–Alkali soil	CK	6456.8 ± 20.3	15.0 ± 2.4	8.7 ± 2.4	12.7 ± 1.7	219.3 ± 7.7	93.2 ± 2.5	68.6 ± 2.2
	AMF	7870.0 ± 14.2 *	19.7 ± 0.3 *	11.3 ± 0.8 *	15.7 ± 0.8 *	248.7 ± 6.3 **	101.8 ± 1.5 *	71.7 ± 0.5 *
LX Normal soil	CK	7502.1 ± 15.9	18.7 ± 1.7	10.3 ± 1.2	14.3 ± 2.4	264.0 ± 20.4	122.9 ± 6.4	72.3 ± 1.4
	AMF	8743.6 ± 36.2 *	23.7 ± 2.5 *	15.7 ± 0.5 *	18.7 ± 0.5 *	284.7 ± 8.4 *	125.7 ± 6.3.0 *	72.5 ± 1.8 ^ns^

^ns^ indicates non-significance; * indicate significant differences at *p ≤* 0.05; ** indicate significant differences at *p ≤* 0.01.

**Table 3:** The physiochemical properties of soil in Laixi and Guangrao experimental sites.

	Organic Matters (g kg^−1^)	Hydrolyzable Nitrogen (mg kg^−1^)	Available Phosphorus (mg kg^−1^)	Available Potassium (mg kg^−1^)	Exchangeable Calcium (g kg^−1^)	Soil Salt Content (g kg^−1^)	pH
LX Normal soil	16.7	89.3	49.6	123.0	2.36	0.8	6.7
GR Saline–alkali soil	6.6	39.5	24.2	93.6	7.52	2.6	8.8

## Data Availability

Not applicable.
